# Impact of inflammation and Treg cell regulation on neuropathic pain in spinal cord injury: mechanisms and therapeutic prospects

**DOI:** 10.3389/fimmu.2024.1334828

**Published:** 2024-01-29

**Authors:** Chunjia Zhang, Yan Li, Yan Yu, Zehui Li, Xin Xu, Zuliyaer Talifu, Wubo Liu, Degang Yang, Feng Gao, Song Wei, Liang Zhang, Han Gong, Run Peng, Liangjie Du, Jianjun Li

**Affiliations:** ^1^ School of Rehabilitation, Capital Medical University, Beijing, China; ^2^ Department of Spinal and Neural Functional Reconstruction, China Rehabilitation Research Center, Beijing, China; ^3^ Institute of Rehabilitation medicine, China Rehabilitation Research Center, Beijing, China; ^4^ School of Population Medicine and Public Health, Chinese Academy of Medical Sciences/Peking Union Medical College, Beijing, China; ^5^ Cheeloo College of Medicine, Shandong University, Jinan, Shandong, China

**Keywords:** spinal cord injury, neuropathic pain, Treg cells, inflammation, cell therapy

## Abstract

Spinal cord injury is a severe neurological trauma that can frequently lead to neuropathic pain. During the initial stages following spinal cord injury, inflammation plays a critical role; however, excessive inflammation can exacerbate pain. Regulatory T cells (Treg cells) have a crucial function in regulating inflammation and alleviating neuropathic pain. Treg cells release suppressor cytokines and modulate the function of other immune cells to suppress the inflammatory response. Simultaneously, inflammation impedes Treg cell activity, further intensifying neuropathic pain. Therefore, suppressing the inflammatory response while enhancing Treg cell regulatory function may provide novel therapeutic avenues for treating neuropathic pain resulting from spinal cord injury. This review comprehensively describes the mechanisms underlying the inflammatory response and Treg cell regulation subsequent to spinal cord injury, with a specific focus on exploring the potential mechanisms through which Treg cells regulate neuropathic pain following spinal cord injury. The insights gained from this review aim to provide new concepts and a rationale for the therapeutic prospects and direction of cell therapy in spinal cord injury-related conditions.

## 1 Introduction

Spinal cord injury (SCI) occurs when there is injury or damage to the spinal cord due to an external force that may result in neurological dysfunction. This injury can affect, to varying degrees, the sensory, motor, and autonomic functions of the body (1). Such injuries result in severe deterioration in the quality of life of patients and increase disability and mortality rates for spinal cord injuries (2, 3). Trauma accounts for approximately 90% of spinal cord injuries (4). Neuropathic pain is a complex disorder caused by neurological lesion or disease and has become a major prognostic challenge for clinical patients due to its difficult-to-treat and often ineffective treatment options (5). A new definition of neuropathic pain was proposed by a panel of experts in 2008: ‘pain that occurs as a direct consequence of an injury or disease affecting the somatosensory system’ (6), which has since been endorsed by the International Association for the Study of Pain (IASP) (7). Peripheral or central neurological lesions can result in loss of sensation in the innervated areas of the damaged nerves or in areas of the body that correspond to areas of the spinal cord or brain that have been directly or indirectly damaged as a result of the lesion or disease. Therefore, sensory hypersensitivity in the affected area is often accompanied by sensory loss when most neuropathic pain occurs (8). Pain resulting from SCI can affect the patients’ quality of life and severely impact the prognosis, which can result in lifelong consequences. Neuropathic pain (NP), a complex and heterogeneous disorder, affects approximately 8% of the adult population and has significant implications for both patients and healthcare systems (9). The International Association for the Study of Pain (IASP) defines NP as pain that arises directly from a lesion or disease affecting the somatosensory system (10). The etiology of NP can be attributed to damage to either the peripheral nerves, resulting in peripheral neuropathic pain (PNP), or the central nerves, resulting in central neuropathic pain (CNP). PNP is commonly associated with conditions such as Complex Regional Pain Syndrome (CRPS) and Failed Back Surgery Syndrome (FBSS), including cancer and diabetes. CNP typically arises following a stroke, spinal cord injury, or multiple sclerosis (11). Around 8% of cases of central pain syndromes manifest in post-stroke patients (12), while spinal cord injury patients constitute approximately 30-50% of cases (13), and those suffering from multiple sclerosis comprise around 20-25% (14). NP is characterized by spontaneous pain (pain that occurs without provocation, such as burning sensations and tingling), allodynia (pain resulting from non-harmful stimuli), and hyperalgesia (an increased response to painful stimuli). Pain after SCI can manifest itself in a variety of ways, and as scar tissue recedes, chronic pain emerges, limiting the prognosis of SCI and affecting neuroplasticity (15, 16). The damage to nerve fibers and neurons following SCI can also cause chronic symptoms of neuropathic pain, which may be closely related to the neuroinflammatory response (17). Neuropathic pain is pain due to nerve fiber damage or chronic compression, which can manifest as a tingling, burning, or electric shock-like pain (8). It is an abnormal pain response due to damage to the nervous system, which may manifest as hypersensitivity or spreading of pain (18). Nerve fiber degeneration or sustained compression on nerve fibers can give rise to neuropathic pain, attributed to aberrant firing or heightened release of neurotransmitters. The propagation of nerve impulses becomes irregular, and these abnormal transmissions can lead to distortion or amplification of pain sensations (19). Subsequent to a spinal cord injury, certain neurons may exhibit augmented excitability, resulting in an excessive amplification of pain signaling. This abnormal excitability might involve neurons beyond the injury site, causing pain sensations to radiate into unaffected areas. The heightened neuronal activity engenders impaired nerve conduction, thereby disrupting the transmission of pain information through the central nervous system (20). A neuroinflammatory response occurs, characterized by tissue swelling and increased pressure in the vicinity of the nerves. Consequently, inflammatory mediators like tumor necrosis factor, prostaglandins, and cytokines are released, thereby leading to aberrant pain perception (8, 21, 22). Alterations and remodeling of neural circuits in the central nervous system manifest following spinal cord injury. This, in turn, can elicit a painful response to otherwise innocuous stimuli, consequently inducing neuropathic pain characterized by sensations of tingling, burning, and electric shock-like pain (8, 23).

Following SCI, the inflammatory response is complex and is driven by a variety of cellular and signaling molecules, including inflammatory factors and injury-associated molecules (e.g., high mobility group protein (HMGB1), heat shock protein (HSP), etc.). These inflammatory mediators subsequently recruit and activate immune cells to further exacerbate the inflammatory response (24). Intervention of the inflammatory response leads to inflammatory cell infiltration, neuronal degeneration, and abnormal neurotransmitter release, which in turn exacerbates the perception and development of neuropathic pain (25). Microenvironmental imbalance and parenchymal cell infiltration are key to secondary SCI (26, 27). The immune response is involved in post-injury microenvironmental regulation; its regulatory role is achieved through interactions with other immune cells, which can regulate the activation state of immune cells and control the intensity of the inflammatory and immune response through cell contact and cytokine secretion (28, 29).

Regulatory T (Treg) cells, a specialized subpopulation of immunosuppressive T cells, are essential for maintaining immune homeostasis (30). There is mounting evidence indicating the involvement of the adaptive T-cell immune response, within the immune system, in the development of neuropathic pain. Previous investigations have demonstrated the infiltration of T cells into the spinal cord (31, 32), the site of injury (33), and the dorsal root ganglion (DRG) subsequent to peripheral nerve injury (34). These studies highlight the indispensable role of T cell regulation in neuropathic pain. Although the precise contributions of distinct T cell subpopulations to neuropathic pain remain unclear, it has been observed that helper T (Th)1 cells are capable of augmenting pain sensitivity through the production of inflammatory cytokines, namely interferon gamma (33). Conversely, helper T (Th)2 cells have shown the ability to diminish pain sensitivity in animal models of nerve injury by generating anti-inflammatory cytokines, such as IL-10 (33). Simultaneous investigations have also suggested a potential link between microglia-mediated gender dimorphism in pain and Treg-mediated regulation of microglia activation and attenuation of pain hypersensitivity (35). Treg cells exert a localized immunosuppressive effect by targeting immune cells to reduce the inflammatory response and decrease self-attack (36). Regulation of Treg cell number and function is an important part of the pathogenesis of various immune diseases. Treg cells act as key negative regulators of inflammation in various pathological states, including autoimmunity, injury, and neurodegeneration (37–43). Regulatory T cells (Tregs) play a crucial role in maintaining self-tolerance and the dynamic equilibrium of the immune microenvironment (44). Given their involvement in immunoregulation during the inflammatory response to neuropathic pain, it is imperative to examine the functions of inflammation and Treg cell regulation in the context of neuropathic pain following spinal cord injury.

**Figure 1:**
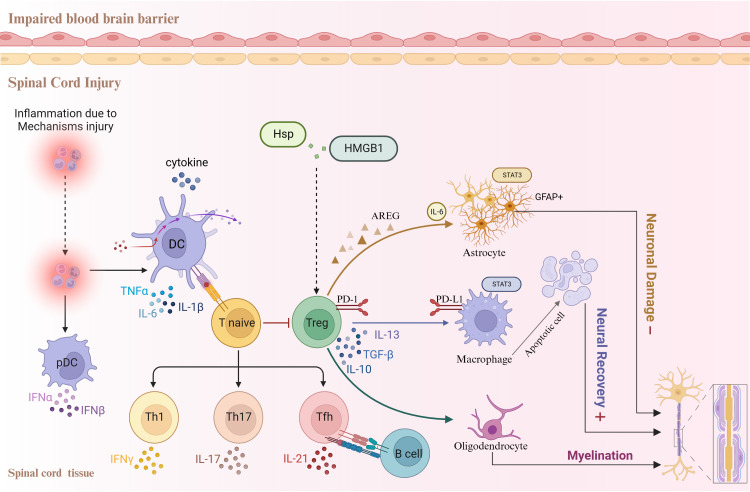
Relationships between the inflammatory response, Treg cells, and other cell types following spinal cord injury.

This review describes the relationship between the inflammatory response and Treg cell regulation following SCI (**Figure 1**), as well as the critical role of Treg cells in the development of neuropathic pain after SCI. The overarching objective of this review is to gain profound insights into the underlying mechanisms of neuropathic pain following SCI and to provide novel avenues for cellular therapeutic interventions.

## 2 The inflammatory response is associated with neuropathic pain after SCI

### 2.1 Release of inflammatory mediators and inflammation-mediated pain transmission

The release of inflammatory mediators and inflammation-mediated pain transmission play an important role in the progression of disease after SCI (45). During the inflammatory response, immune cells and nerve cells interact to trigger the release of inflammatory mediators, including cytokines (tumor necrosis factor (TNF)-α, interleukin (IL)-1β) and chemokines (e.g., CXCL1, CXCL2). These inflammatory mediators are involved in the inflammatory response and regulate inflammation-related signaling pathways (46, 47).

In addition, the release of inflammatory mediators participates in the inflammation-mediated pain transduction process. Following SCI, inflammatory mediators stimulate sensory neurons and dorsal root ganglion cells, leading to increased neuronal excitability (48). This abnormal state of excitability induces pain perception, which is mediated through signaling pathways, during which inflammatory mediators act as signaling molecules that interact with their corresponding receptors and ion channels to regulate neuronal excitability (49, 50). Such receptors and channels include TRPV1 channels, ATP receptors, and acid-sensing ion channels. Inflammatory mediators alter neuronal excitatory thresholds and enhance neuropathic pain by modifying the activity and expression of these channels (51–54). Therefore, inflammatory mediators are involved in pain transmission following SCI.

### 2.2 Effect of the infiltration and activation of immune cells on pain after SCI

**Figure 2:**
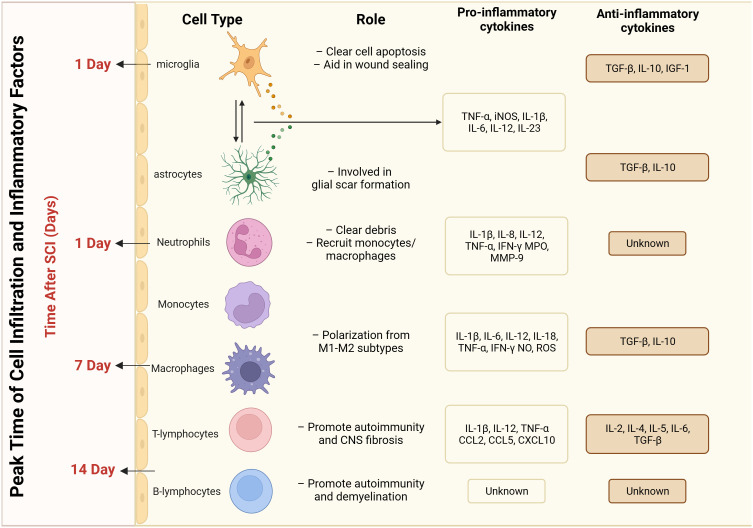
Peak time of infiltration and inflammatory factors released by different cell types after spinal cord injury.

SCI elicits an immune response, resulting in the accumulation of inflammatory and immune cells. Immune cell infiltration occurs at the site of SCI where immune cells interact with neurons (55, 56). These infiltrating immune cells mainly include monocytes, macrophages, T cells, and B cells (57). Different cell types release different immune molecules at the site of injury after SCI, and the peak period of cellular infiltration varies (**Figure 2**). Immune cells are activated to release a series of cytokines and chemicals, including TNF-α, IL-1β, and IL-6, which an act directly on neurons to increase excitability and decrease inhibition (58). The interaction between immune cells and nerve cells leads to increased neuronal excitability, triggering or enhancing the nociceptive transmission of pain through direct stimulation of neurons or via effects on synaptic transmission (59). Related studies have shown that inhibiting the infiltration and activation of immune cells, or targeting and modulating cytokines and chemicals released by immune cells, can attenuate pain sensation following SCI (60–63). Therefore, modulation of the infiltration and activation of immune cells may be a novel approach to treat pain after SCI.

### 2.3 The role of glial cells in the inflammatory response and the maintenance of neuropathic pain

Glial cells, including astrocytes and microglia, are involved in the inflammatory response following SCI (64–66). These glial cells become activated after SCI and release multiple inflammatory mediators (67, 68) (**Figure 2**). Primary injury is initiated by an initial insult to the spinal cord, leading to mechanical damage and subsequent opening of the blood-brain barrier (BBB). This process is characterized by oxidative damage, edema, ischemia, and heightened glutamate excitability (25, 69). Within the initial few hours, these mechanisms contribute to the onset of secondary damage, whereby immune cells infiltrate the damaged region via the vascular system, resulting in cell death and exacerbated injury. Various cell types are involved in this secondary phase, exerting distinct temporal influences on disease progression (25, 70). Spinal cord injury induces the activation and recruitment of multiple glial cells, leading to intricate downstream effects on neuronal function (71). The formation of the glial scar after SCI involves the participation of various cell types. Astrocyte activation begins on day 1 post-injury and reaches its peak at day 14 (72–75). Schwann cell recruitment starts 21 days after SCI (76, 77). Meningeal cells become involved 3 days after SCI and reach their peak at day 14 (78–80). Fibroblast activation initiates 3 days after SCI and peaks at day 7-14 (81–84). Finally, a limited degree of structural tissue regeneration and repair takes place in the weeks to months following spinal cord injury (25). Astrocytes, the most common glial cell type in the spinal cord, play an important role in maintaining normal neuronal function, regulating the blood–brain barrier, and removing intercellular metabolites (85, 86). The deleterious effects of astrocytes during SCI are produced through reactive astrocytes. Two types of reactive astrocytes have been identified: the A1 astrocyte and the A2 astrocyte (87, 88). The former plays a destructive role and promotes the inflammatory response, whereas the latter plays a restorative role in ischemia-induced inflammation and inhibits the inflammatory response (89). The imbalance between the A1 and A2 responses is an important mechanism in the development of neuropathic pain after SCI. Excessive A1 astrocyte responses and excessive release of inflammatory mediators may lead to neuronal activation and abnormal nociceptive transmission, resulting in the development of neuropathic pain (90). The A2 astrocyte response is a major contributor to the development of neuropathic pain. In addition, deficiencies in the A2 astrocyte response may affect tissue repair and anti-inflammatory mechanisms, perpetuating the inflammatory response and exacerbating the degree and duration of pain (91).

Microglia are mainly found in the gray matter regions of the central nervous system. Following SCI, microglia are also activated and participate in the inflammatory response, promoting neuronal excitability and inflammation-mediated nociceptive transmission (92). Microglia act as powerful neuromodulators to regulate salience transmission and pain transmission through multiple inflammatory mediators (e.g., pro-inflammatory and anti-inflammatory factors) acting on neurons and other glial cells (93) (**Figure 2**). Microglia have multiple cell surface receptors that dynamically and multifacetedly regulate the inflammatory response after SCI by interacting with neurons, astrocytes, immune cells, and others (94). Studies have reported that microglia are involved in inflammatory responses, pain signaling, and synaptic remodeling after SCI. Microglia maintain the inflammatory response and the enhancement of pain afferent signaling through the recruitment of immune cells after injury, forming synaptic structures with neurons. Vesicles released through these structures enhance neuronal excitability and strengthen pain signaling (95–98). Furthermore, it has been reported that the HMGB1–RAGE axis contributes to the major macrophage/microglia-mediated pro-inflammatory response, and that inhibition of this pathway exerts neuroprotective functions after SCI. This cascade modulation of the immune microenvironment has emerged as a prospective therapeutic approach for the treatment of SCI (99). In addition to the well-studied microglia and astrocytes, oligodendrocytes, as the main myelin-producing glial cells, are critical in maintaining myelin for fast and efficient conduction of electrical impulses along the axon and for maintaining axon integrity (100). Some studies have reported that Treg cells are involved in oligodendrocyte differentiation and myelination, which has a positive effect on SCI recovery (101).

## 3 Regulatory role of Treg cells in SCI

### 3.1 Function and characterization of Treg cells

Treg cells are an immunosuppressive subpopulation of CD4+ T helper cells, which have important immunomodulatory functions and unique characteristics that help to maintain immune homeostasis and inhibit overactivation of the immune response (102, 103). The functions of Treg cells are characterized by immunosuppression, immune tolerance, and immune homeostasis.

Recent advances in Treg cell biology have identified Treg cells residing in specific tissues for the maintenance of tissue homeostasis and repair (104), such as in the secondary prevention of ischemic stroke where they suppress immune responses by directly inhibiting the activation and function of other immune cells (105). Treg cells play an important role in the immune system as self-tolerance regulators, preventing damage to tissues from the immune response and reducing the occurrence of autoimmune diseases. Furthermore, Treg cells are involved in tumor development and progression by suppressing tumor immunity; Treg cells can be activated by chemokines (e.g., CCR4-CCL17/22, CCR8-CCL1, CCR10-CCL28, and CXCR3-CCL9/10/11), are chemotactically attracted to the tumor microenvironment, and participate in microenvironment regulation (106–108).

The dysregulation of the Th17 and Treg cell balance in neurological disorders can significantly impact disease progression (39). Excessive activation of Th17 cells and insufficient regulation by Treg cells can contribute to immune-mediated neuroinflammation and injury, thereby promoting disease progression (109, 110). Additionally, emerging evidence highlights the interconnectedness of the gut, spinal cord, and immune cells in spinal cord injury disorders, which establishes a “gut-spinal cord-immune” axis. Treg cell regulation in the intestinal environment, along with the promotion of IL-10 secretion, can modulate the dynamic equilibrium between Treg and IL-17γδ T cells, suppress inflammatory responses, and enhance motor function recovery in rats. Collectively, these studies underscore the crucial regulatory function of Treg cells in the pathogenesis of spinal cord injury (111).

CD25 is one of the hallmark features of Treg cells. Increased expression of CD25 by Treg cells is closely associated with immunosuppressive functions (112). In addition, Treg cells express the transcription factor FOXP3 in the nucleus, which is a major marker of Treg cell identity and a key molecule in the regulation of their function (113, 114). Transforming growth factor β (TGF-β) and IL-10 are examples of the multiple inflammatory suppressive cytokines produced by Treg cells; these factors inhibit the activation and immune response of other cells, leading to immunosuppression and immunomodulation (115, 116). Short-chain fatty acids (SCFAs) modulate Treg cells in the gut and affect the balance of Treg cells and IL-17+γδ T cells in the spinal cord, which has been reported to suppress inflammatory responses and promote locomotor function in SCI rats (111). Treg cells interact with amphiregulin (AREG) through the AREG/epidermal growth factor receptor (EGFR) signaling pathway to participate in immune regulation while controlling skeletal muscle function and regeneration (117). In summary, Treg cells play an important role in immune regulation by suppressing the activation and function of other immune cells, maintaining immune homeostasis, and preventing damage to host tissues from the immune response.

### 3.2 Treg cell regulation of the inflammatory response after SCI

A reduction in the number of Treg cells is closely associated with the inflammatory response following SCI. Treg cells regulate the degree of inflammation through a variety of mechanisms; they can inhibit the activity of other immune cells (e.g., Th1, Th2, and Th17 cells) and reduce the production and release of inflammatory mediators (118, 119). The number of Treg cells was reported to be significantly reduced in the spinal cord and its surrounding tissues, which resulted in a decrease in the immunomodulatory function of Treg cells, allowing an over-activated inflammatory response to develop at the site of injury (103). Treg cells reduce the extent of the inflammatory response by inhibiting the activation of other immune cells and the production of inflammatory factors. The inflammatory response in SCI is directly suppressed by inhibiting the activation signaling of other immune cells through the binding of the surface molecules cytotoxic T lymphocyte antigen 4 (CTLA4) and programmed cell death 1 (PD-1) to their ligands (120, 121). Treg cells act as regulatory antigen presenters through specific ligand–receptor interactions, inhibiting antigen activation and inflammatory responses by immune cells (120). Treg cells also interfere with inflammatory signaling pathways, and can reduce the production of inflammatory factors and inflammatory responses by inhibiting the activation of the nuclear transcription factor NF-κB (122–124). In addition, some studies have reported that by regulating the TUG1/miR-214/HSP27 signaling pathway, the proportion of Treg cells can be reduced, thereby alleviating acute SCI (125). Treg cells can also regulate neuroendocrine circuits by inhibiting cytokine secretion and release; therefore, regulating neuronal activity Janyga et al., 2023^1^. Treg cells downregulate TNF-α and IL-6 expression in microglia by inhibiting STAT3 pathway activation, which ultimately improves the damaged spinal cord microenvironment and promotes the recovery of neurological function after SCI (126). MBP-Th2 cell transplantation after SCI changes the state dominated by Th1 and M1 cells to a state dominated by Th2, Treg, and M2 cells. This changes the local immune microenvironment by increasing the number of Th2 cells, thus producing beneficial effects on the spinal cord and promoting the repair of SCI.

### 3.3 Inhibition of neuropathic pain by Treg cells

Neuroimmune communication has emerged as a key neuropathic pain mechanism in previous studies, which reported that both the innate and adaptive immune systems are associated with neuroinflammatory changes in neuropathic pain (127). Local infiltration of macrophages, T cells, astrocytes, and activated microglia following SCI results in the release of multiple inflammatory mediators (pro-inflammatory cytokines, such as TNF-α, IL-1β, IL-17, and IFN-g) to maintain nociceptive hypersensitivity (128–131) (**Figure 2**). In mice, mechanical pain after nerve injury can be alleviated by intrathecal injection of Treg cells (132). Furthermore, CD28 agonists can alleviate mechanical pain hypersensitivity due to injury in rats with chronic compression injury of the sciatic nerve by modulating the number of T cells, macrophages, and other immune cells in the sciatic nerve and dorsal root ganglion (44, 133). The elimination of Treg cells using a CD25 antibody leads to prolonged mechanical abnormalities in sciatica mice (44). Depletion of FoxP31 Treg cells in transgenic DEREG mice leads to a transient increase in mechanical pain hypersensitivity (134).

Treg treatment also modulates the amount of reactive astrocytes of different phenotypes to reduce neurotoxicity by attenuating astrocyte GFAP expression. Interestingly this therapeutic effect of Treg cells manifests itself differently in female and male mice, with a reduction in the number of neurotoxic astrocytes in peripherally injured male mice, and conversely, in peripherally injured females, the number of protective astrocytes was increased with peripheral nerve injury (135). Brain Treg cells inhibit neurotoxic astrocyte proliferation and protect neurons from damage by producing the low-affinity EGFR ligand AREG (136). Male mouse meningeal Treg cell administration may induce an anti-inflammatory shift in microglia phenotype via Treg-associated effector cytokines IL-10 and TGF-β (137–139).

The inhibitory role of Treg cells in neuropathic pain has been confirmed by a series of animal studies, as described above. The results of clinical trials similarly suggest that the number of T helper cells producing IL-17 is reduced, while the number of Treg cells is increased in patients with chronic lower back pain. Correspondingly, mRNA expression levels of FOXP3 and TGF-β were elevated in peripheral blood mononuclear cells according to cytokine profiling assays (140, 141). This phenomenon has been speculated to reduce pain levels in patients through the suppression of inflammatory responses (142). In summary, Treg cells reduce inflammatory damage to neurons by modulating neuroimmune interactions, and reducing neuronal hyperexcitability and abnormal alterations in synaptic plasticity, which in turn attenuates the onset and progression of neuropathic pain.

## 4 Clinical application strategies for Treg cell enhancement and synergy

### 4.1 Therapeutic strategies for Treg cell increase and functional improvement

The number of Treg cells can be increased by exogenous donor acquisition or endogenous proliferation and expansion, for example, with the use of growth factors, immunomodulators, or cell therapy, among others (143–145). Targeting Treg cell ligand–receptor interactions using specific cytokines or drugs (e.g., IL-10, TGF-β, and IL-2, etc.) that enhance Treg cell immunosuppression, enhancing immunomodulation, improves the function of Treg cells (146, 147). Improving Treg cell migration and accurately localizing damaged sites by altering chemical gradients, enhancing cytokine adhesion, and modulating inflammatory factor expression is one of the important therapeutic strategies (148–150).

### 4.2 Synergistic effects of Treg cells with immunosuppressants and other treatments

Treg cells, as natural immunomodulatory cells, can inhibit immune cell activity, and can function synergistically with immunosuppressive agents (e.g., immunosuppressant drugs or cytokine inhibitors) to reduce inflammatory and autoimmune responses (151–153). Cell therapy for SCI is emerging as a new research hotspot (154). Currently common cell therapies, such as stem cell therapy or gene therapy, can repair and regenerate tissues. Treg cell combined with stem cell therapy is a potentially more desirable therapeutic tool, and the synergistic combination of drugs with Treg cells enhances the immune-suppressive effect of the drugs, reduces the side effects, and lowers the risk of immune tolerance (155). Treg deficiency affects the gut microbiota and bile acid metabolism, induces IL-6 expression, and triggers a lethal inflammatory response. Antibiotics can modulate the gut microbiota and bile acid metabolism by inhibiting IL-6 levels, thus preventing the lethal inflammation caused by Treg deficiency (156). In studies on immune-related diseases associated with tumors, Treg cells are implicated in tumor development and progression by suppressing anti-tumor immune responses. Therefore, there is a critical need in the field of cancer immunotherapy to deplete Treg cells and modulate their function to enhance anti-tumor immune responses (106). Research has demonstrated that dendrobine significantly reduces Foxp3 expression, increases serum IL-17 levels, and enhances Th17 cell function while suppressing Treg cell function. Additionally, *in vivo*, dendrobine and cisplatin may synergistically regulate the Treg/Th17 cell balance rather than induce apoptosis (157). All-trans retinoic acid is involved in regulating the differentiation of helper T cells (Th) and Treg cells. Furthermore, all-trans retinoic acid maintains the stability of thymus-derived Treg cells under inflammatory conditions (158). Severe asthma development is particularly associated with Th17 cell and neutrophil activation, and studies have shown that asthma patients can effectively suppress airway inflammation by increasing the Treg/Th17 cell ratio using statins in combination with corticosteroids (159, 160). Tregs are key target cells involved in asthma relief, and it has been suggested that glucocorticoid application reduces the number and activity of Tregs in various asthma mouse models, potentially through thymic T-cell production inhibition (161). Immunomodulators (e.g., anti-CD3 antibody, anti-CD25 antibody, etc.) can directly influence the number and activity of Treg cells, and targeted depletion of Treg cells can activate tumor-specific effector T cells and enhance the efficacy of tumor immunotherapy (162). Cancer immunotherapy primarily focuses on immune checkpoint molecules, and blocking CTLA-4 primarily activates T cells and suppresses Treg cells. PD-L1 plays a dominant role in Th1 and Th17 immunity, while PD-L2 primarily impacts Th2 immunity (163). The use of Treg cells as a cell-based therapeutic approach was initially demonstrated in mouse models; Treg cells were found to have a beneficial role in pathogenesis (36). However, the immune rejection faced by this therapeutic approach is considered to be one of the important challenges.

## 5 Prospects and challenges in the application of Treg cell therapy

From the current degree of clinical application, firstly, there still exists a certain technical difficulty in large-scale preparation of high-purity Treg cells. Second, further clinical research and validation are still needed to determine the therapeutic mechanism and safety of Treg cells. In addition, the survival time and functional stability of Treg cells also requires further improvement. Overall, although there are still some challenges and limitations in the clinical application of Treg cells, they have great potential in regulating inflammatory responses and treating immune-related diseases. Therefore, Treg cells are expected to be a potential target for regulating inflammatory responses and treating neuropathic pain after SCI, which will bring better therapeutic effects and treatment strategies for SCI patients.

## 6 Conclusions

Spinal cord injury (SCI) is a severe neurotraumatic condition that frequently results in the development of neuropathic pain. Neuropathic pain following spinal cord injury (SCI) is a complex spectrum of disorders characterized by a multitude of pathophysiologic mechanisms and associations with psychosocial factors, posing significant challenges in its management (164). While research in recent decades has shed light on the pathophysiology of neuropathic pain after SCI, therapeutic advancements have been limited. Given the high prevalence of chronic neuropathic pain, future research will prioritize the investigation of targeted therapies, identification of reliable biomarkers, and evaluation of combination therapies targeting multiple mechanisms to enhance treatment efficacy. Inflammation is known to play a critical role in the early stages following SCI, but excessive inflammation can exacerbate painful symptoms. Treg cells have a pivotal function in regulating inflammation and reducing neuropathic pain. Treg cells regulate inflammatory responses by influencing cytokine expression and other immune cell functions. However, inflammation also hinders the activity of Treg cells, thus exacerbating neuropathic pain. Therefore, besides suppression of the inflammatory response, enhancing the regulatory function of Treg cells may also offer new therapeutic avenues for the treatment of neuropathic pain caused by SCI. It is very valuable and meaningful to study the potential regulatory function of Treg cells in neuropathic pain after spinal cord injury or even central nervous system injury. Future research on neuropathic pain after spinal cord injury may focus on the development of new immunomodulatory drugs, assessment of the number and function of patients’ Tregs cells to form a personalized treatment plan, the development of vaccines to regulate the immune system, and novel cell therapies based on the *in vitro* expansion of Tregs technology and the transfer of Tregs cells into the patient’s body. However, it is important to note that the clinical application of Tregs for the treatment of neuropathic pain requires a careful consideration of human Treg cell purity, stability, and functional role in neuropathic pain disorders.

## Author contributions

CZ: Data curation, Formal Analysis, Investigation, Methodology, Resources, Supervision, Validation, Writing – original draft. YL: Formal Analysis, Investigation, Writing – review & editing. YY: Formal Analysis, Investigation, Resources, Writing – review & editing. ZL: Software, Writing – review & editing. XX: Data curation, Formal Analysis, Writing – review & editing. ZT: Resources, Writing – review & editing. WL: Supervision, Visualization, Writing – review & editing. DY: Conceptualization, Writing – review & editing. FG: Project administration, Writing – review & editing. SW: Software, Writing – review & editing. LZ: Data curation, Writing – review & editing. HG: Methodology, Writing – review & editing. RP: Validation, Writing – review & editing. LD: Visualization, Writing – review & editing. LJ: Conceptualization, Data curation, Funding acquisition, Investigation, Methodology, Resources, Validation, Visualization, Writing – review & editing.

## Footnotes

1 Janyga S, Kajdaniuk D, Czuba Z, Ogrodowczyk-Bobik M, Urbanek A, Kos-Kudła B, et al. Interleukin (IL)-23, IL-31, and IL-33 play a role in the course of autoimmune endocrine diseases. *Endocr Metab Immune Disord Drug Targets*. (Ahead of Print) (2023). doi: 10.2174/1871530323666230908143521
